# Tribological Behavior of Nanolubricants Based on Coated Magnetic Nanoparticles and Trimethylolpropane Trioleate Base Oil

**DOI:** 10.3390/nano10040683

**Published:** 2020-04-05

**Authors:** José M. Liñeira del Río, Enriqueta R. López, Manuel González Gómez, Susana Yáñez Vilar, Yolanda Piñeiro, José Rivas, David E. P. Gonçalves, Jorge H. O. Seabra, Josefa Fernández

**Affiliations:** 1 Laboratory of Thermophysical Properties, Nafomat Group, Department of Applied Physics, Faculty of Physics, Universidade of Santiago de Compostela, 15782 Santiago de Compostela, Spain; josemanuel.lineira@usc.es (J.M.L.d.R.); enriqueta.lopez@usc.es (E.R.L.); 2 Applied Physics Department, NANOMAG Laboratory, Faculty of Physics, Universidade de Santiago de Compostela (USC), 15782 Santiago de Compostela, Spain; manuelantonio.gonzalez@usc.es (M.G.G.); susana.yanez@usc.es (S.Y.V.); y.pineiro.redondo@usc.es (Y.P.); jose.rivas@usc.es (J.R.); 3 Institute for Science and Innovation in Mechanical Engineering and Industrial Engineering (INEGI), Universidade do Porto, Dr. Roberto Frias St., 4200-465 Porto, Portugal; degoncalves@inegi.up.pt; 4 Faculty of Engineering of the University of Porto (FEUP), Dr. Roberto Frias St., 4200-465 Porto, Portugal; jseabra@inegi.up.pt

**Keywords:** friction, wear, film thickness, magnetic nanoparticles and nanolubricant

## Abstract

The main task of this work is to study the tribological performance of nanolubricants formed by trimethylolpropane trioleate (TMPTO) base oil with magnetic nanoparticles coated with oleic acid: Fe_3_O_4_ of two sizes 6.3 nm and 10 nm, and Nd alloy compound of 19 nm. Coated nanoparticles (NPs) were synthesized via chemical co-precipitation or thermal decomposition by adsorption with oleic acid in the same step. Three nanodispersions of TMPTO of 0.015 wt% of each NP were prepared, which were stable for at least 11 months. Two different types of tribological tests were carried out: pure sliding conditions and rolling conditions (5% slide to roll ratio). With the aim of analyzing the wear by means of the wear scar diameter (WSD), the wear track depth and the volume of the wear track produced after the first type of the tribological tests, a 3D optical profiler was used. The best tribological performance was found for the Nd alloy compound nanodispersion, with reductions of 29% and 67% in friction and WSD, respectively, in comparison with TMPTO. On the other hand, rolling conditions tests were utilized to study friction and film thickness of nanolubricants, determining that Fe_3_O_4_ (6.3 nm) nanolubricant reduces friction in comparison to TMPTO.

## 1. Introduction

In 2014, the worldwide energy consumption was around 396 EJ for different uses such as transport, residential consumption or industrial activity [1]. In all these sectors, mechanical systems suffer energy losses, mostly due to friction and wear, and therefore their reduction is decisive. Thus, in economic terms the annual total losses originating from tribological contacts are estimated to be 2,536,000 million euros, being 73% due to friction and 27% due to wear [2]. Friction and wear losses can be reduced substantially by using new tribological solutions. In recent years, nanotechnology has been introduced in the development of numerous applications due to the physical and chemical properties of the nanomaterials being quite different from those of the bulk materials [3,4]. Recently several articles showed that the addition of nanoparticles (NPs) to current lubricants of mechanical elements can considerably reduce both friction and wear [4,5,6,7]. The main advantages of using NPs as additives with respect to other materials are due the higher capabilities to reduce friction and wear and even to repair the worn surface. This is due to the small size of NPs that allows them to enter the contact area, resulting in a positive lubrication effect [8]. Moreover, the nanoparticles used as additives can be less chemically reactive than the common additives since the NP films are formed mechanically, so they are more durable and less reactive than those with other additives [9]. In addition, NPs interact less with other additives present in a lubricant and since their film formation is largely mechanical, they may form films on many different types of surface [9] Another of the advantages of using NPs as lubricant additives is their low volatility which avoid losses at high temperature conditions [9]. Accordingly, NPs not only provide the nanolubricants with higher anti-wear and anti-friction capabilities and but also a higher ability for heat dissipation. NPs as friction modifier additives can work by different lubrication mechanisms: rolling effect and tribofilm formation, owing to the direct effect of the nanoparticle on the surface, and mending and polishing effects, due to surface enhancement [10].

Stribeck curves are used to explain how nanoparticles play a role in friction over elasto-hydrodynamic lubrication (EHL), mixed lubrication (ML) and boundary lubrication (BL) regimes. Normally, boundary lubrication occurs under low-speed and high-load conditions, so nanolubricant additives are essential in this lubrication regime due to the high friction coefficient in these conditions [11,12]. The improvement produced by the nanoparticles is due to their high affinity for the metal surfaces, so they adhere to them preventing the metal–metal contact. Zin et al. [13] studied the tribological properties through Stribeck curves of nanolubricants formed by an engine oil and carbon nano-horns as additives. These authors conclude that nanolubricants containing carbon nano-horns exhibit better anti-friction properties in all the different lubrication regimes (boundary, mixed and elasto-hydrodynamic lubrication). Furthermore, Ghaednia et al. [14] analyzed the tribological behavior of nanolubricants also through Stribeck curves. These lubricants consist of CuO nanoparticles in mineral base oil using sodium oleate as a surfactant. These authors deduced that CuO additives decrease the friction coefficient deeply into the boundary lubrication regime in comparison to the mineral base oil.

Despite the numerous studies and advances that have been made with nanoparticles in the field of lubricants, there is still a serious problem with the stability of the nanodispersions against sedimentation, since nanoparticles tend to agglomerate with each other [15]. It is well known that dispersants have been used in order to increase the stability time of nanoparticles [16,17,18,19]. Better results can be obtained modifying the nanoparticles’ surface by reacting with a surfactant. The resulting nanoparticles are known as coated or functionalized nanoparticles. Chen et al. [15] recently reviewed the time stability of numerous nanolubricants. For this task, these authors analyzed different characteristics of nanoparticles such as material nature, particle size, and surface modification, concluding that surface modification is essential to disperse nanoparticles into a lubricating oil. This modification can be done mainly through reaction with surfactants [20] or alkoxysilanes [21] as surface modifiers.

Carbon-based and metallic-based nanoparticles are two of the most utilized nanomaterials for anti-friction and anti-wear applications [22,23,24,25]. Magnetic metal nanoparticles are broadly studied due to their numerous applications such as magnetic records, drug-delivery agents and anti-wear additives of lubricants, among others [26]. In the case of lubricants, there are several articles about iron oxide nanoparticles with good anti-friction and anti-wear properties: Hu et al. [16] analyzed the tribological properties of non-coated ferric oxide (Fe_2_O_3_, particle size around 20–50 nm), used as an additive in a lubricating mineral oil (SN500) using sorbitol monostearate as dispersing agent. These authors observed that the anti-wear properties of the base oil were improved slightly (11.6%) by the addition of Fe_2_O_3_. Zhou et al. [26] analyzed the tribological behavior of coated Fe_3_O_4_ magnetic nanoparticles (particle size around 10 nm) with oleic acid dispersed in a liquid paraffin, showing that the coefficient of friction and the wear can be effectively reduced up to 25% and 65%, respectively, with the addition of the nanoparticles. Recently, Zhang et al. [27] studied the tribological performance of PAO 6 (a polyalphaolefin, whose kinematic viscosity at T = 373.15 K is around 6 mm^2^ s^-1^) with graphene oxide/Fe_3_O_4_ nanocomposites as additives obtaining reductions of 7% and 52% in friction and wear, respectively, in comparison to PAO 6.

The aim of this work is to analyze the tribological behavior in different lubrication regimes of nanolubricants composed by different types and sizes of functionalized superparamagnetic nanoparticles with an oleic acid coating. For this purpose, Fe_3_O_4_ with sizes of 6.3 and 10 nm and Nd alloy compound with average sizes of 19 nm and trimethylolpropane trioleate (TMPTO) were chosen. This biodegradable base oil is a non-flammable hydraulic fluid, which is known as a good boundary lubricant [28] with a high viscosity index [29]. In addition, there is expected to be a good affinity between the trioleate group of the oil and the oleic coating of the nanoparticles. There are no previous studies about the anti-friction and anti-wear properties of the Nd alloy compound nanoparticles as a lubricant additive. For this work, ball-on-three-plates tests were performed at 20 °C to determine the friction coefficient at boundary conditions as well as to quantify the wear by means of a 3D profiler. Moreover, the wear track surface was analyzed through confocal Raman microscopy in order to know the role that nanoparticles play in the reduction of the wear. For mixed and elastohydrodynamic lubrication regimes, Stribeck curves were measured in a ball-on-disk tribometer under rolling conditions at operating temperatures of 30, 50 and 80 °C. These conditions were selected because rolling elements usually work at temperatures around 80 °C while the behavior at low temperatures can occur if the equipment needs to make a cold start. Additionally, film thickness measurements of prepared nanolubricants were performed at different temperatures in order to analyze their lubrication capacity. There are no previous research works on Stribeck curves or film thickness measurements with ferrites as additives of lubricants. Rolling bearing tests were also performed for the coated Fe_3_O_4_ (6.3 nm) nanolubricant.

## 2. Materials and Methods

### 2.1. Base Oil

The TMPTO base oil sample was provided by Croda (Snaith, United Kingdom). A high-performance liquid chromatograph (HPLC) coupled to a quadrupole orthogonal acceleration time-of-flight mass spectrometer micrOTOFQ™ (Bruker Daltonics Inc., Billerica, MA, USA) equipped with an electrospray ionization source (ESI) was used to characterize the sample. The procedure used is the same as that previously reported for a different lot [30]. The analysis shows that the base oil used in the present work is composed by 69.2% of trimethylolpropane trioleate (Figure S1), 26.5% of a very similar compound with a C–C bond more than TMPTO (i.e., two H atoms less) and 4.3% of another compound with two extra C–C bonds (i.e. four H atoms less). 

### 2.2. Synthesis and Characterization of Nanoparticles

#### 2.2.1. Chemical and Materials

All chemicals used were of analytical grade and without purification. Iron (III) acetylacetonate (Fe(acac)_3_), 1,2-hexadecanediol, oleic acid, phenyl ether, cyclohexane, tri-n-octylamine (TOA), ferrous sulfate (FeSO_4_·7H_2_O), hydrochloric acid (HCl) and ammonium hydroxide (NH_4_OH) were purchased from Sigma (Saint Louis, MO, USA). Oleylamine was obtained from Acros Organics (Geel, Belgium). Ethanol was purchased from Panreac (Madrid, Spain) and ferric chloride (FeCl_3_·6H_2_O) was obtained from Alfa Aesar (Madrid, Spain).

#### 2.2.2. Synthesis

Two different sets of Fe_3_O_4_@Oleic_acid NPs were prepared according to a modification of Sun’s method [31] in order to produce samples with tailored size, with average diameters around 6 nm and 10 nm. The oleic acid was used as a surfactant to stabilize the NPs.

To produce Fe_3_O_4_@Oleic_acid NPs with an average diameter around 6 nm (S1), 10 mmol of Fe(acac)_3_, 50 mmol of 1,2-hexadecanediol, 30 mmol of oleic acid, 20 mmol of oleylamine, and 100 mL of phenyl ether, were mixed and magnetically stirred under a flow of nitrogen. The mixture was heated to 250 °C for 4 h before being cooled down to room temperature. After the addition of ethanol to induce the precipitation of Fe_3_O_4_ nanoparticles, the individually formed dispersed nanoparticles were magnetically separated and washed several times with ethanol (4×) and cyclohexane (3×). Finally, NPs were resuspended in cyclohexane. 

In the case of the coated Fe_3_O_4_@Oleic_acid NPs with average diameter of 10 nm (S2), a coprecipitation procedure following Massart´s method [32] was employed with some modifications. Fe_3_O_4_ NPs of 10 nm, the coprecipitation method was used, according to the Massart´s method with some modifications. A solution containing 45 mmol of FeCl_3_·6H_2_O and 30 mmol of FeSO_4_·7H_2_O and 100 mL of HCl (0.01 M) were mechanically stirred (250 rpm) and heated to 60 °C. Then, 750 mmol of NH_4_OH and 8 mmol of oleic acid were added to obtain the hydrophobic character of the magnetic nano-particles and the reaction was stirred for 1 h. Afterwards, the mixture was transferred to a beaker and placed on a hot plate at 100 °C to allow for precipitation. The magnetic nanoparticles obtained were washed with Milli-Q water (3×) and were resuspended in cyclohexane.

The Nd alloy compound was prepared by thermal decomposition. Typically, 6 mol of Nd oleate were added to a flask containing oleic acid and TOA. After being filled with nitrogen atmosphere, the flask was then heated at 330 °C for 72 h. Nanoparticles were precipitated magnetically separated and washed with ethanol (4×) and cyclohexane (4×). Finally, the NPs were resuspended in cyclohexane.

**Figure 1:**
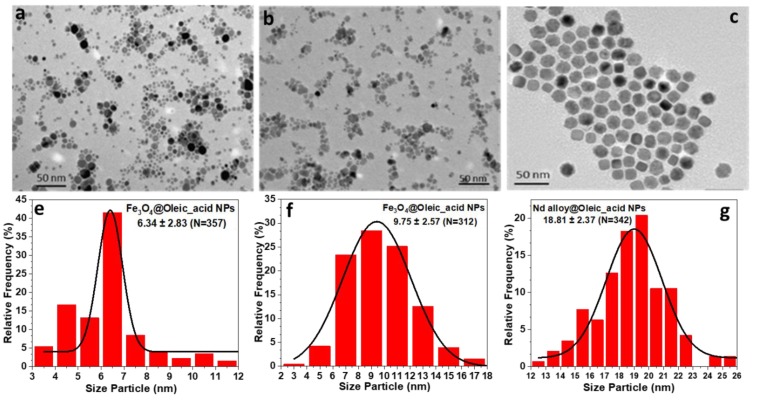
(**a**–**c**) Transmission electron microscopy (TEM) micrographs and (**e**–**g**) size distributions of the Fe_3_O_4_@Oleic acid with average sizes of 6.3 nm (**a** and **e**); 10 nm (**b** and **f**) and Nd alloy compound with size of 19 nm (**c** and g) nanoparticles. The size distribution was performed using Image J software.

All prepared nanoparticles were fully characterized with different techniques. The morphology and size of coated Fe_3_O_4_ and Nd alloy compound (Figure 1) were determined by transmission electron microscopy (TEM), using a JEOL JEM-1011 microscope operating at 100 kV (JEOL, Tokyo, Japan). The micrographs of Fe_3_O_4_ and Nd alloy compound NPs are presented in Figure 1a–c, together with their corresponding histograms from Figure 1e to Figure 1g. It can be clearly observed that Fe_3_O_4_@Oleic_acid NPs have a spherical core, are individually dispersed, and show a moderate size distribution with averaged sizes around 6.3 nm, in one case, and 10 nm in the other case. Nd alloy compound nanoparticles have a nearly cubic shape with narrow size distribution and an average size around 19 nm.

**Figure 2:**
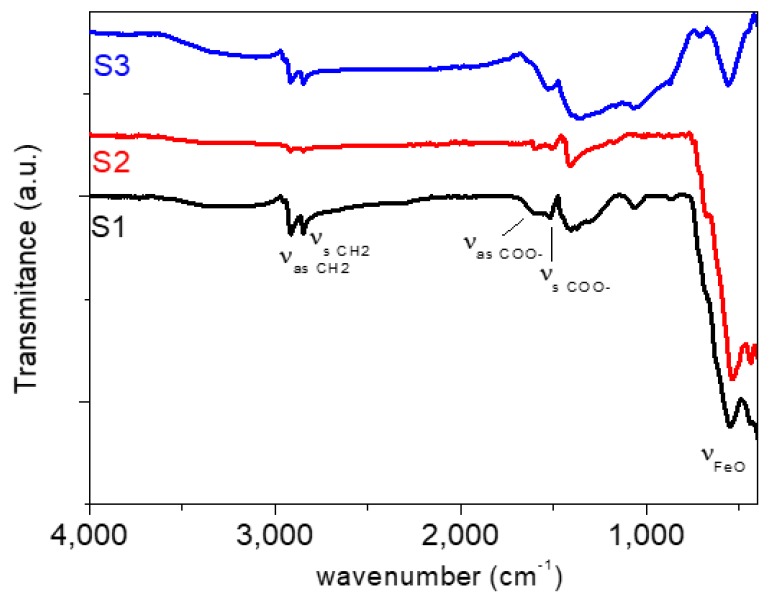
Fourier transform infrared spectroscopy (FT-IR) spectra of coated Fe_3_O_4_ nanoparticles: (S1) Fe_3_O_4_ (6.3 nm), (S2) Fe_3_O_4_ (10 nm) and (S3) Nd alloy compound (19 nm).

The Fourier transform infrared spectroscopy (FT-IR) technique was used to characterize the nanoparticles using a Thermo Nicolet Nexus spectrometer (Thermo Fisher Scientific, Madrid, Spain) and the attenuated total reflectance (ATR) method in the range 400–4000 cm^−1^. As can be seen in Figure 2, the FT-IR spectra of coated Fe_3_O_4_ nanoparticles present the characteristic bands of oleic acid groups and Fe_3_O_4_. For both samples, an absorption peak appears around 572 cm^−1^ which corresponds to the Fe-O vibration of the magnetic Fe_3_O_4_ phase [33]. Two absorption peaks can be observed at 2925 and 2848 cm^−1^ that can be attributed to the asymmetric and symmetric CH_2_ stretching in oleic acid, respectively [33,34]. Besides, the CH_3_ umbrella mode appears around 1406 cm^−1^ [34]. In addition, two peaks appear around 1598 and 1521 cm^−1^ which correspond to the asymmetric and symmetric stretching vibrations of COO− groups of the oleic acid [34]. Furthermore, the wavenumber separation (∆) between the *V*_as_(COO−) and *V*_s_(COO−) bands can be used to study the type of the interaction (monodentate, bridging bidentate, chelating bidentate or ionic interaction) between the carboxylate head and the metal atom [35]. In this case, the ∆ of 77 cm^−1^ is ascribed to a chelating bidentate interaction between the COO^−^ group and the Fe or Nd atoms. This result is in concordance with previous works, in which a strong chemical bond between carboxylic acid and the amorphous iron oxide or neodymium alloy nanoparticles’ surface has been shown [36,37].

**Figure 3:**
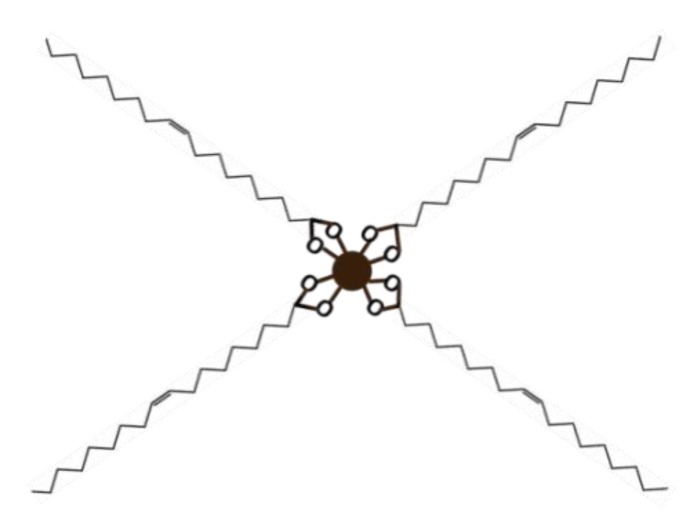
Representation of the oleic acid-particle structure.

Also, thermogravimetric analysis (TGA) was used to estimate the oleic acid content in the synthesized nanoparticles as well as the number of oleic acid molecules per surface area of each nanoparticle (Table 1). For this aim, a Perkin Elmer Pirys 7 TGA (Perkin, Waltham, MA, USA) was employed heating from 50 to 850 °C at 10 °C/min under a nitrogen flow of 20 mL/min. Figure 3 shows a schematic representation of the oleic acid-particle structure.

**Figure 4:**
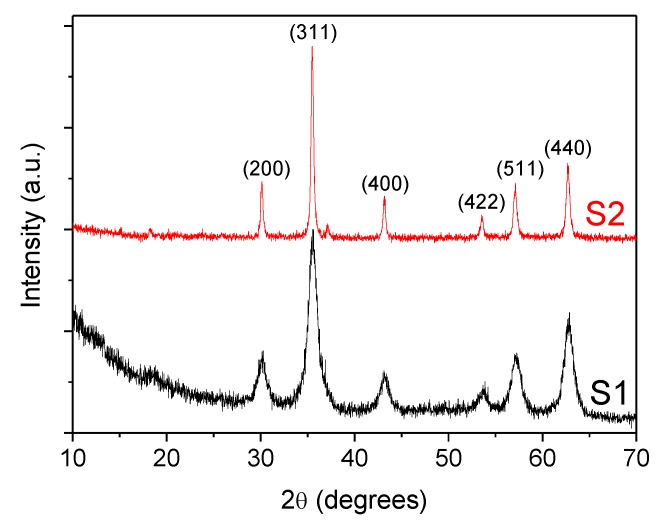
X-ray powder diffractogram (XRD) for the coated F_3_O_4_ nanoparticles: (S1) 6.3 nm and (S2) 10 nm.

Additionally, crystallinity and the phase structures of coated Fe_3_O_4_ nanoparticles were also characterized by X-ray powder diffraction (XRD) using a Philips PW1710 diffractometer (Cu Kα radiation source, λ = 1.54186 Å) with a step size of 0.02° and counting time of 2 s per step from 10 to 80° (2θ). In Figure 4, the diffractograms of both samples S1 and S2, show a set of peaks corresponding to those of the crystalline Fe_3_O_4_ phase (Joint Committee on Powder Diffraction Standards, JCPDS 79-0417) [36]. Moreover, the widening of the peaks, indicate the ultra-fine nature and small crystallite size of the particles [37]. An additional broad band appearing at low angles 2θ< 20° is related to the presence of oleic acid. The Nd alloy compound is formed by a mixture of NdFe_12_B_6_ and Fe_3_O_4_.

**Figure 5:**
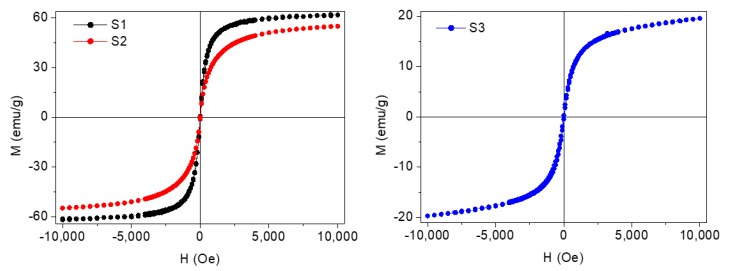
Magnetization curves at room temperature for the coated F_3_O_4_ nanoparticles: (S1) 6.3 nm and (S2) 10 nm and (S3) Nd alloy.

Magnetization of the coated NPs was also measured. For this task, dried samples were magnetically characterized using a vibrating sample magnetometer (VSM) (DMS/ADE Technologies, Massachusetts, USA) at room temperature with applied magnetic fields between −10 and 10 kOe. In Figure 5 the typical hysteresis loops of the coated nanoparticles, normalized to their magnetite mass content, can be observed. It was concluded that these species were superparamagnetic owing to their almost zero coercivity and zero remanence in the magnetization curve. Saturation magnetization (M_S_) of these Fe_3_O_4_ coated NPs (S1 and S2) is lower than bulk magnetite saturation (~92 emu/g). This fact is in accordance to that expected for the reduction of magnetization caused by a dead magnetic layer in small magnetic nanoparticles and the non-magnetic contribution of the oleic acid coating shell. The magnetization of Nd alloy NPs (S3) shows a nearly superparamagnetic behavior and a saturation magnetization around 20 emu/g.

### 2.3. Preparation of the Nanolubricants

In order to obtain homogeneous nanolubricants, the nanoparticles suspended in cyclohexane after the synthesis were added to the oil through the elimination of the solvent by boiling with a rotary evaporator. For this aim, the first step was to find a solvent which can be easily and completely separated from the oil. Different organic solvents with lower boiling point than that of the TMPTO base oil, such as cyclohexane, ethanol, ether or chloroform, were tested. For this purpose, TMPTO was mixed with each one of these solvents and then was separated with the rotary evaporator at different temperature conditions. In order to know whether the solvent was completely removed, the viscosity of the TMPTO obtained was measured with a rotational viscometer Stabinger SVM3000 (Anton Paar, Graz, Austria) and compared with that of the neat TMPTO. Chloroform was the solvent for which the viscosity of the base oil remained unchanged. Hence, it has been concluded that the best solvent to incorporate the nanoparticles in TMPTO is chloroform. Therefore, the nanoparticles that after synthesis were dispersed in cyclohexane were transferred to chloroform. Subsequently, concentration is determined by thermogravimetry and the chloroform dispersion was added to the TMPTO base oil and mixed by ultrasonic agitation for 15 min. Then, the chloroform was eliminated from the oil using a rotary evaporator. Using this procedure, three nanodispersions of TMPTO with 0.015 wt% of each NP were obtained. Once all the solvent has been removed, the dispersion of oil and nanoparticles are sonicated with a homogenizer Fisherbrand ultrasonic bath, in a continuous shaking mode for 240 min with an effective power of 180 W and a fixed sonication frequency of 37 kHz to obtain the nanolubricants. Density and viscosity of the nanolubricants and TMPTO base oil measured with the Stabinger SVM3000 deviceare shown in Table 2 at reference temperatures and Tables S1 and S2 from 278.15 K to 373.15 K. For this device, the expanded uncertainties (k = 2) are 0.02 K for the temperature from 288.15 to 378.15 K and 0.05 K outside this range, 0.0005 g·cm^−3^ for density and 1% for dynamic viscosity.

### 2.4. Tribological Behavior: Ball-on-Three-Pins Test

Tribological tests were carried out under pure sliding conditions in a modular compact rheometer MCR 302 from Anton-Paar equipped with a tribology cell T-PTD 200, in combination with a Peltier hood H-PTD 200 for a precise temperature control. This rheometer has an outstanding speed and torque control, an accurate normal force detector as well as a fast temperature control in a wide range. All these characteristics are utilized for the tribology tests. In this work, the test configuration used was ball-on-three-pins. The ball is fixed on a shaft and driven by the MCR rheometer motor, then rotating on the three pins under the fixed normal force. The resulting torque is associated with the friction force by using simple geometric calculations. The axial force of the rheometer is transferred into a normal force acting perpendicular to the bottom pins at the contact points. Zero distance is determined by a ‘zero-gap measurement’, in which the measuring system (ball) is lowered by the rheometer until softly touching the sample surface (i.e., a normal force will be detected). The height at which the normal force is sensed corresponds to zero height. The distance resolution is better than 0.01%. More details can be found in the literature [38]. The diameter of the ball is 12.7 mm, the cylindrical-shaped pins have 6 mm in both diameter and height. Balls and pins were cleaned with a stream of hexane and dried with air before the tests. Samples were fully submerged by adding 1.3 mL of lubricant. The ball rotates on the three pins with a total normal force of 45 N, resulting in a force of 21 N acting normally to each pin surface during the tests, which corresponds to a maximum contact pressure of about 1 GPa and a mean contact pressure around 0.7 GPa [39]. Tests were performed at a constant rotational speed 213 rpm (corresponding to a linear speed 0.1 m/s) and a sliding distance of 340 m at 20 °C. Three replicates were run for each concentration of lubricant. 

In order to analyze the worn surface after the ball on three-pins tests, a 3D Optical Profiler Sensofar S Neox was used to quantify the wear of pins in different terms such as wear scar diameter (WSD), wear track depth (WTD) and the volume of the wear hole. In order to obtain representative average values, these parameters have been measured in three different zones using a confocal mode with a 10× objective. Furthermore, a WITec alpha300R+ confocal Raman microscopy was used to obtain information about the composition in the pins worn track.

Roughness (*Ra*) of worn surfaces of pins lubricated with the studied nanolubricants and the base oil were also analyzed to characterize the anti-wear capability of the nanolubricants with this device. For this purpose, the ISO4287 standard (International Organization for Standardization, Vernier, Switzerland) was used employing a Gaussian filter with a long wavelength cut-off of 0.08 mm. 

### 2.5. Film Thickness Measurements

A ball-on-disc tribometer, EHD2 model from PCS Instruments, equipped with optical interferometry, was employed in order to measure the lubricant film thickness of the contact formed between a steel ball (19.05 mm in diameter) and a rotating glass disc (coated with a 20 nm chromium and 500 nm silica layer). The disc and ball are driven by two electric motors for carrying out tests under rolling/sliding conditions, the load-applying system being based on moving the ball against the disc. Thus, the normal load is measured with a load cell, positioned below the ball carriage, measuring the normal load applied on the ball against the disc. The friction force is also measured on the ball, through a torque cell mounted on the ball shaft with the disc rotating faster than the ball, and afterwards, at the same entrainment/rolling speed, the friction force is measured again with the ball rotating faster than the disc. Therefore, the friction coefficient is then calculated from the normal force and the friction force. The central film thickness is obtained by optical interferometry through the wavelength of the light returned from the central plateau of the contact [40]. The glass disc can be tested up to approximately 0.7 GPa of maximum Hertz pressure and the ball specimen presents a high-grade surface finish. The ball and disc characteristics (provided by the manufacturer) are presented in Table 3. 

The zero distance is evaluated before each film thickness test. The ball is loaded against the disc (same load as the test load 50 N), without lubricant between the disc and the ball. The space layer coating thickness is then evaluated through interferometry. The corresponding wavelength is recorded and set as the zero point. Measured wavelengths above that value means that the film thickness is higher than zero, and below that value mean the space layer is worn out.

Film thickness measurements were carried out under fully flooded lubrication (130 mL of lubricant oil) for the base oil and the magnetic nanolubricants at three operating temperatures (30, 50 and 80 °C) using a load of 50 N which corresponds to a maximum Hertz pressure of 0.66 GPa and for 5% slide-to-roll ratio (SRR) defined as:

(1) $$\mathrm{SRR}\left(\%\right)=2\times \frac{\left(U_{\mathrm{disc}}-U_{\mathrm{ball}}\right)}{\left(U_{\mathrm{disc}}+U_{\mathrm{ball}}\right)}\times 100,$$

being *U_disc_* and *U_ball_* the speed of disc and ball on the contacting surfaces respectively.

The entrainment speed (*U_s_*) is defined by the following equation:

(2) $$U_{S}=\frac{\left(U_{\mathrm{disc}}+U_{\mathrm{ball}}\right)}{2},$$

For each temperature the same entrainment speed ramp was used: 0.01 m/s to 2 m/s. These conditions allow a very thin lubricant film thickness to be worked with. The lowest ball speed is 0.097 m/s and the highest ball speed is 1.950 m/s for the three studied temperatures. On the other hand, the lowest disc speed is 0.102 m/s and the highest disc speed is 2.049 m/s. The tribometer adjusts disc and ball speeds automatically, being the disc speed faster in order to get positive SRR and the disk slower to obtain negative SRR, while the entrainment speed is maintained constant. The result is the average of three measurements, two ramps increasing speed and one decreasing.

### 2.6. Tribological Behavior: Stribeck Curves

Friction coefficient measurements were also performed with the EHD2 ball-on-disc described in the previous section. For these measurements, the ball runs against a steel disc under 50 N load producing contact pressures up to 1.11 GPa. The used balls and discs are made of carbon steel (American Iron and Steel Institute, AISI 52100 100Cr6) with 19.05 mm and 100 mm diameters, respectively. The disc properties were provided by the manufacturer and the surface roughness was measured with a Hommelwerke Profiler for both polished and rough samples (Table 3), while the characteristics of the ball are the same as for the lubricant film thickness measurements. Friction coefficients of nanolubricants and base oil were also measured at 30, 50 and 80 °C. For both discs (rough and polished) the friction properties have been studied through Stribeck curves for a 5% SRR value and entrainment speeds from 0.01 to 2 m/s. For this test, the range of the ball and disc speeds and the SSR are the same as for the film thickness measurements. The friction coefficient values are also given by the average of measurements with 5% SSR.

### 2.7. Rolling Bearing Test Rig

To carry out the rolling bearing tests a modified four-ball machine by Marques et al. [41] was employed. The machine configuration was replaced by a rolling bearing assembly in order to test a different kind of rolling bearings [41]. This device consists mainly of two different parts, the upper part is directly connected to the machine shaft and the second part is where the bearing system is fitted, 50 mL of sample volume assures that the lubricant level reaches the middle of the rollers. In this work, SKF 51107 thrust ball bearings have been used. The friction torque and operating temperature at different points were continuously measured during the tests; the first one was measured with a piezoelectric torque cell Kistler 9339, which ensured high-accuracy measurements even at very low friction torque [41,42]. The temperature was measured by three thermocouples in real time at different strategic locations: inside the oil sump, near the rolling bearing raceway and the lubricant, and the third measured the room temperature. More details were reported by Marques et al. [41].

The machine initially worked at 500 rpm for 10 min at very low load (5 kg applied in a dead weight lever system, the resulting force being approximately 1000 N) in order to ensure the warm-up of the system. Subsequently, an axial load of 7 kN was applied, producing a maximum contact pressure of around 2.3 GPa in each ball contact. At the same time the heater was switched on to reach the required oil bath temperature of 70 °C. Subsequently, five friction torque measurements were carried out for each of the five speed steps (100, 200, 500, 1000 and 1500 rpm) for each lubricant in order to obtain better repeatability. Friction torque measurements were taken after 30 min once the chosen speed, load and temperature had been reached. 

## 3. Results

### 3.1. Stability of the Nanolubricants

**Figure 6:**
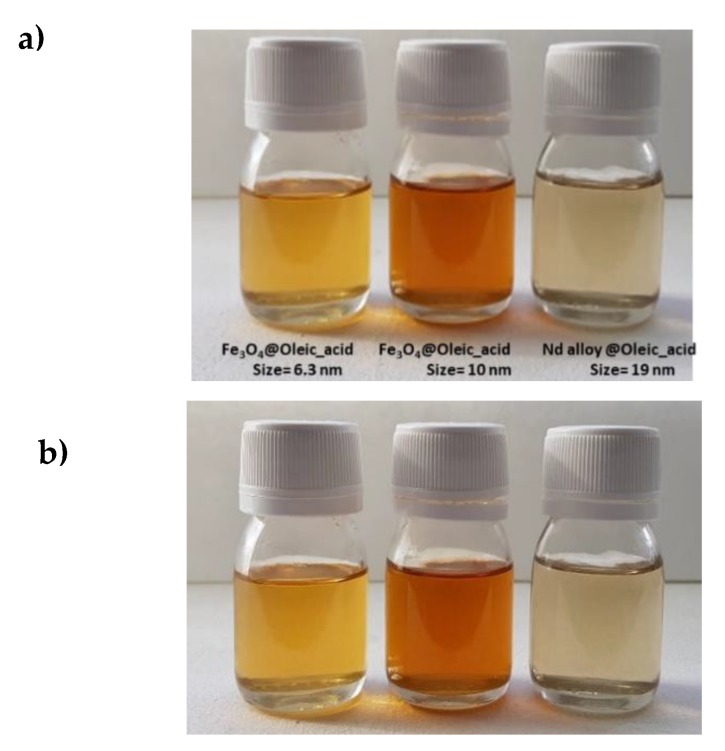
Visual observation of the stability of the nanolubricants TMPTO + 0.015 wt% NP: (**a**) just after sonication of 240 min; (**b**) 11 months after sonication.

In a first step, the stability of the nanolubricants against sedimentation was studied by visual observation. For the three prepared nanodispersions no signs of sedimentation appeared for 11 months just after sonication as can be seen in Figure 6. In the Chen et al. [15] review about the stability of numerous nanolubricants, nevertheless, none of them showed time stabilities as large as those for the nanolubricants presented in this work. Indeed, the stability times were much longer than those required to perform ball on three pins and rolling test bearing, 4 and 6 h respectively. 

**Figure 7:**
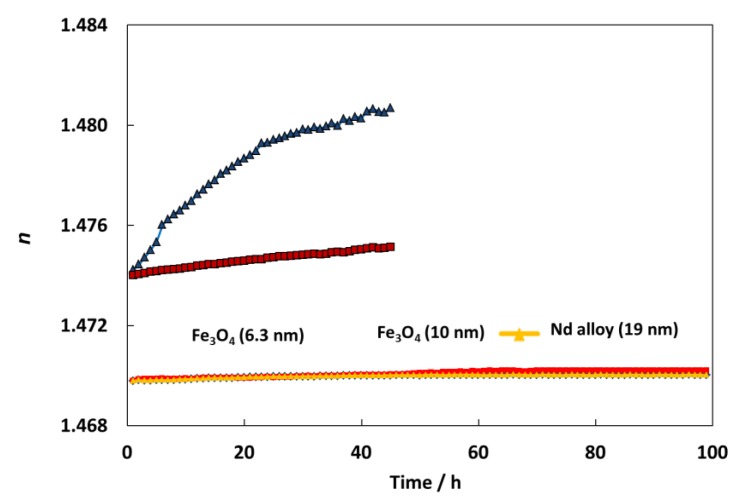
Temporal evolution of the refractive index, *n*, for TMPTO + 0.015 wt% (Fe_3_O_4_ (6.3 nm), Fe_3_O_4_ (10 nm) or Nd alloy (19 nm), TMPTO + 0.05% (graphene oxide, GO or reduced graphene oxide, rGO) [45].

Refractometry is the second method to analyze the stability against sedimentation of prepared nanodispersions. For this aim a Mettler Toledo Refractometer was used to measure the refractive index of nanodispersions over time. This procedure was previously explained [43]. Figure 7 shows an amazing stability over time for all the nanodispersions, showing an important improvement of the stability in comparison with other nanodispersions previously studied [44,45]. Specifically, for TMPTO-based nanolubricants, the refractive index increases 0.44 and 0.08 for graphene oxide, GO and reduced graphene oxide, rGO nanoparticles after 50 h [45]. Meanwhile for this period, in this work the refractive index evolution shows increases of 0.02, 0.03 and 0.02 for Fe_3_O_4_ (6.3 nm), Fe_3_O_4_ (10 nm) and Nd alloy (19 nm) nanolubricants, respectively. These great stabilities can be attributed to the oleic acid coating of the nanoparticles as well as to its affinity with the trioleate group of the TMPTO.

### 3.2. Friction Behavior in Ball-on-Three-Pins

**Figure 8:**
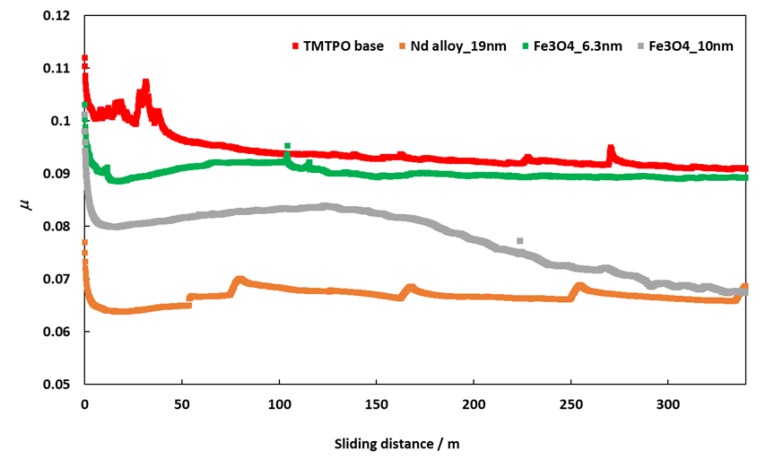
Evolution of friction coefficient values for TMTPO base oil and nanolubricants of TMPTO and 0.015 wt% of each NP with the sliding distance at 20 °C.

**Figure 9:**
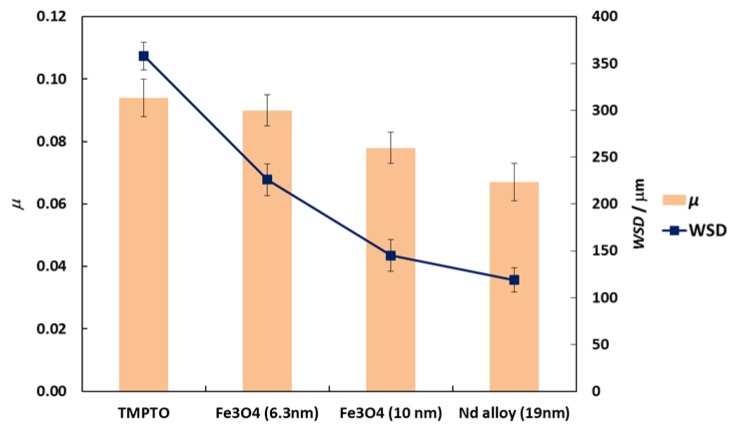
Friction coefficient, *μ*, (■) and WSD, (■) obtained with the neat oil TMPTO and with the three nanolubricants with 0.015 wt% of each NP. Error bars indicate the standard deviation of the mean friction coefficient or of the WSD.

Friction coefficients (μ) obtained with the tribology cell T-PTD 200 from Anton-Paar for the three nanolubricants composed by coated Fe_3_O_4_ (6.3 nm), Fe_3_O_4_ (10 nm) and Nd alloy (19 nm) with TMPTO base oil over time are presented in Figure 8. It can be seen clearly that for the three nanodispersions the friction coefficients obtained are lower than those of the base oil without additives, the greatest friction reduction being obtained for the nanolubricant formed by the Nd alloy nanoparticles in TMPTO base oil. The average friction values are given in Table 4 and plotted in Figure 9. The lowest average friction coefficient is 0.067. This result leads to a friction reduction of 29% due to the Nd alloy additives. In the case of coated magnetites, reductions of 4% and 18% in the average coefficient of friction were obtained for Fe_3_O_4_ (6.3 nm) and Fe_3_O_4_ (10 nm), respectively.

### 3.3. Surface Analysis of Worn Pins

**Figure 10:**
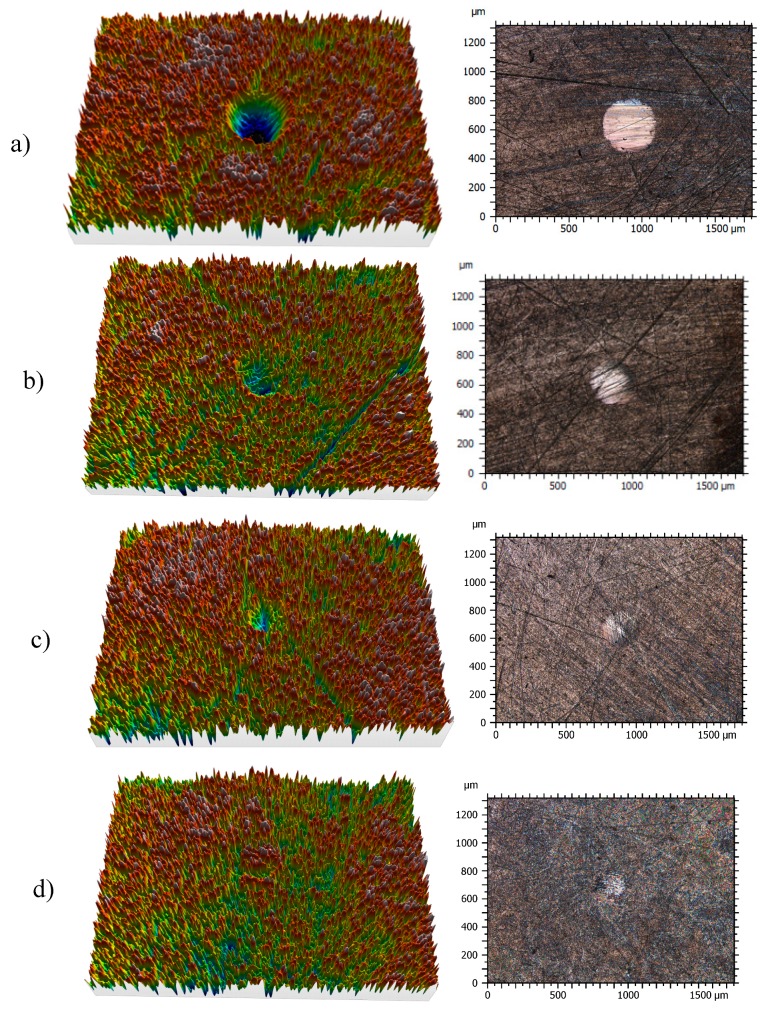
Three-dimensional (3D) profile (10×) and 2D images (10×) of the wear tracks of pins for: (**a**) TMPTO, (**b**) TMPTO + 0.015 wt% Fe_3_O_4_ (6.3 nm), (**c**) TMPTO + 0.015 wt% Fe_3_O_4_ (10 nm) and (**d**) TMPTO + 0.015 wt% Nd alloy (19 nm).

**Figure 11:**
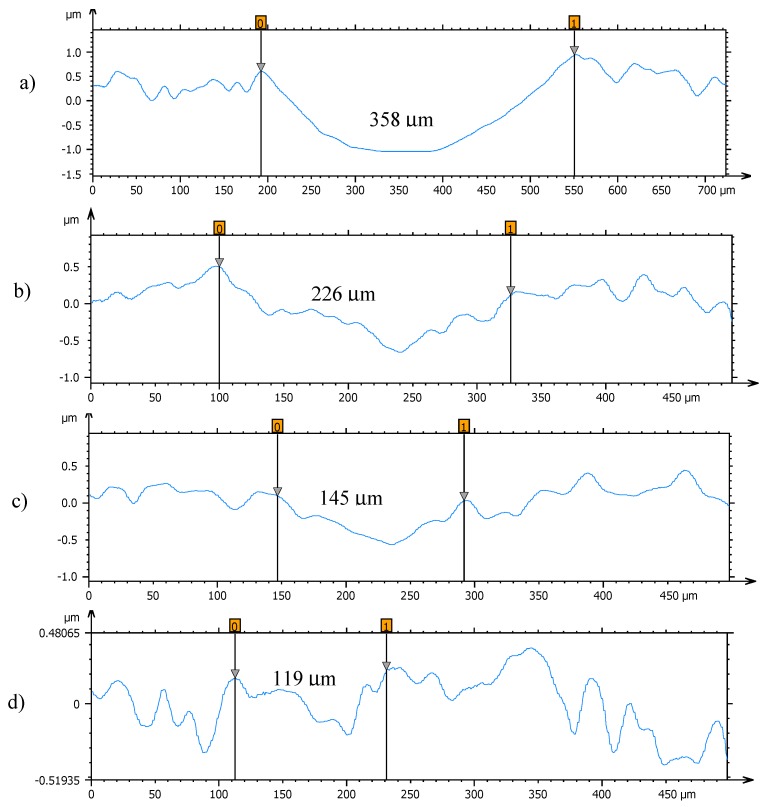
Cross section profiles of the wear tracks on the pins lubricated with (**a**) TMPTO, (**b**) TMPTO + 0.015 wt% Fe_3_O_4_ (6.3 nm), (**c**) TMPTO + 0.015 wt% Fe_3_O_4_ (10 nm) and (**d**) TMPTO + 0.015 wt% Nd alloy (19 nm).

Profiles of the wear tracks produced in the pins due to the ball-on-three-pins tests for the studied nanolubricants and TMPTO oil are shown in Figure 10. The wear in the pins was evaluated through the diameter, depth and volume below the unworn surface of the track (Table 4). Figure 11 shows cross-section profiles of the wear tracks in the pins lubricated with TMPTO base oil and with the nanolubricants. It can be observed that, using the nanolubricants, the obtained wear is lower than for the TMPTO base oil, in terms of diameter, depth and volume of wear scar. For diameter and depth of the wear track, the maximum reductions were obtained with the Nd alloy nanolubricant, being 67% and 35% respectively. For the magnetite nanolubricants, improved results were also observed in comparison with the base oil. Thus, for Fe_3_O_4_ (6.3 nm) reductions of 37% and 3% were observed for diameter and depth of the wear scar respectively whereas for Fe_3_O_4_ (10 nm) reductions of 59% and 25% were obtained respectively. These results show a close relationship between friction and wear behaviors, as it can be seen in Figure 9.

Roughness (*Ra*) of worn surfaces of pins has been also analyzed to characterize the anti-wear capability of the nanolubricants. Table 5 shows that the worn surfaces lubricated with the nanolubricants are smoother than those lubricated with TMPTO. An *Ra* value of 61 nm was obtained for the worn surface lubricated with TMPTO whereas for the track corresponding to the Nd alloy nanolubricant the lowest *Ra* was found (37 nm), which leads to a 54% reduction in roughness. As a result, it can be concluded that a polishing effect occurs due to the presence of nanoparticles. In the case of the Nd alloy this effect seems to be stronger than for the other nanoparticles. As regards magnetites, the smoother surface was obtained with the bigger size of the nanoparticles (10 nm). 

**Figure 12:**
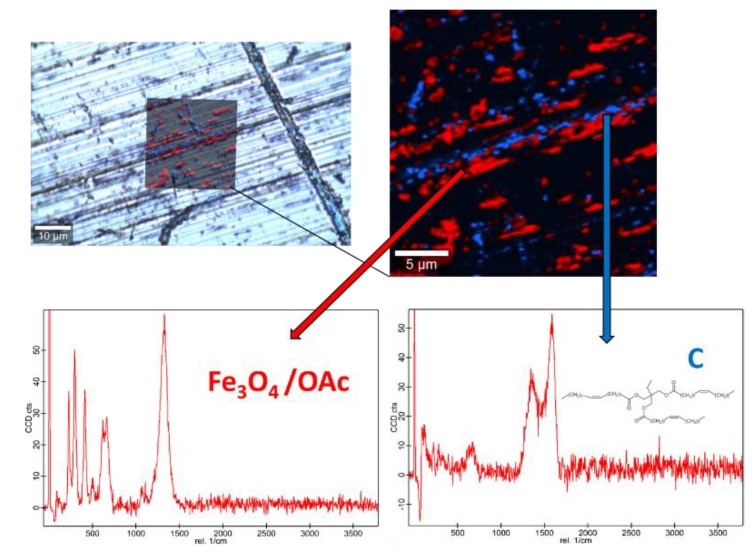
Raman spectra and elemental mapping of the worn surface obtained with the nanolubricant of Fe_3_O_4_ (6.3 nm) nanoparticles.

**Figure 13:**
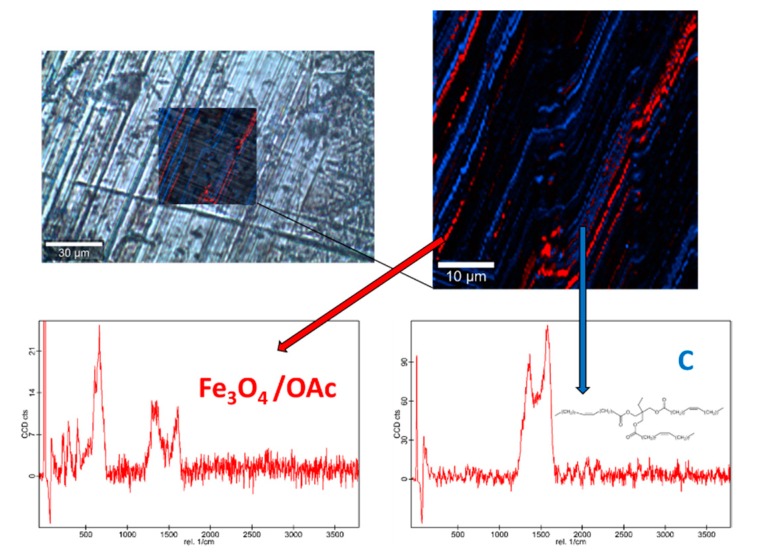
Raman spectra and elemental mapping of the worn surface obtained with the nanolubricant of Fe_3_O_4_ (10 nm) nanoparticles.

**Figure 14:**
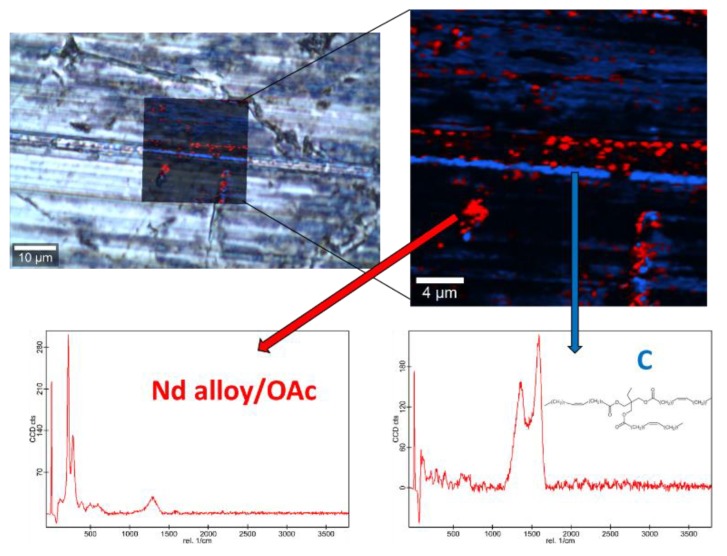
Raman spectra and elemental mapping of the worn surface obtained with the nanolubricant of Nd alloy (19 nm) nanoparticles.

Raman spectra of the base oil (Figure S2) and the three studied magnetic nanopowders (Figures S3 and S4) as well as elemental mapping of the worn pin surfaces lubricated with the three nanolubricants (Figure 12, Figure 13 and Figure 14) were carried out with the confocal Raman microscope at a wavelength of 532 nm to know the role that nanoparticles play in the reduction of surface wear of pins. The Raman spectrum of both magnetites (Figure S4) exhibits characteristic bands at 289 cm^−1^ which is due to the E_g_ phonon mode and at 682 cm^−1^ that is associated to the A_1g_ phonon mode [46]. In addition, the peaks observed at 1580 cm^-1^ and 1309 cm^−1^ are consistent with the presence of the oleic acid coating, specifically these peaks are associated to C=C alkyl stretching and C-H ethylene, respectively [47]. As regards the Nd alloy (Figure S4), there is no previous characterization about this compound.

Figure 12, Figure 13 and Figure 14 show the mapping of worn surfaces lubricated with the nanolubricants containing both magnetites and Nd alloy. Important tribofilms are evidenced owing to a significant presence of nanoparticles (red color). Furthermore, the presence of TMPTO base oil (blue color) is observed. Both magnetite and Nd alloy nanoadditives are mainly placed along several furrows on the worn surface, especially in the case of the Fe_3_O_4_ (10 nm)-based nanolubricant. This fact indicates that a mending effect takes place [43]. Moreover, the major presence of nanoparticles can be observed in Figure 12, which corresponds with the smallest nanoparticle size (6.3 nm). This fact seems to indicate that these nanoparticles have a high affinity to the surface although the tribofilm is less effective at protecting it which is evidenced by the poorer anti-friction and anti-wear performances. Taking into account the Raman and roughness results it can be concluded that the mechanisms which explain the role of these nanoparticles as lubricant additives are the tribofilm formation, which is a direct effect of the nanoparticle on the worn surface, as well as mending and polishing effects which are related to surface enhancement [43].

### 3.4. Film Thickness

**Figure 15:**
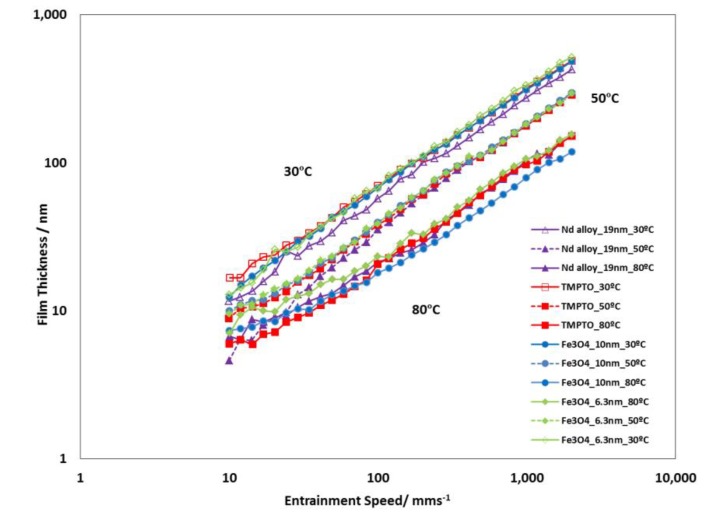
Film thickness of the TMPTO base oil and prepared nanolubricants at slide-to-roll ratio (SRR) of 5%.

Figure 15 shows the typical graph log(film thickness) versus log(entrainment speed). As expected, the higher is the entrainment speed, the thicker is the film. All studied lubricants (base oil and nanolubricants) show similar film thickness values for each operating temperature (30 °C, 50 °C and 80 °C) during the tests performed, due to their very similar viscosities (Table 1 and Table S2). Specifically, the maximum viscosity increase is only 2.8%, which was obtained for the Nd alloy at 278.15 K in comparison to the base oil. Viscosity increase is common when a base oil is additive with nanoparticles [44]. On the other hand, the viscosity, pressure-viscosity coefficient and film thickness of the lubricants decrease when temperature rises [48]. Similar film thickness values of the nanolubricants with respect to those of the base oil is an advantage when it is needed to replace the oil by the nanolubricant in the mechanical elements such as gearboxes or bearings. 

### 3.5. Friction Behaviour: Stribeck Curves

The Stribeck curves for all lubricant samples were studied at temperatures of 30, 50 and 80 °C and SRR of 5%. The coefficient of friction is presented against the specific film thickness, Λ, which in this work, is given by:

(3) $$\Lambda =\frac{h_{0}}{\sigma },$$

where $h_{0}$ is the central film thickness and σ is the average roughness given by $\sigma =\sqrt{{\left(\sigma_{\mathrm{disc}}\right)}^{2}+{\left(\sigma_{\mathrm{ball}}\right)}^{2}}$. Other authors use the minimum film thickness instead to calculate *Λ*, but for the purpose of this analysis, this would only shift the curves to the left.

**Figure 16:**
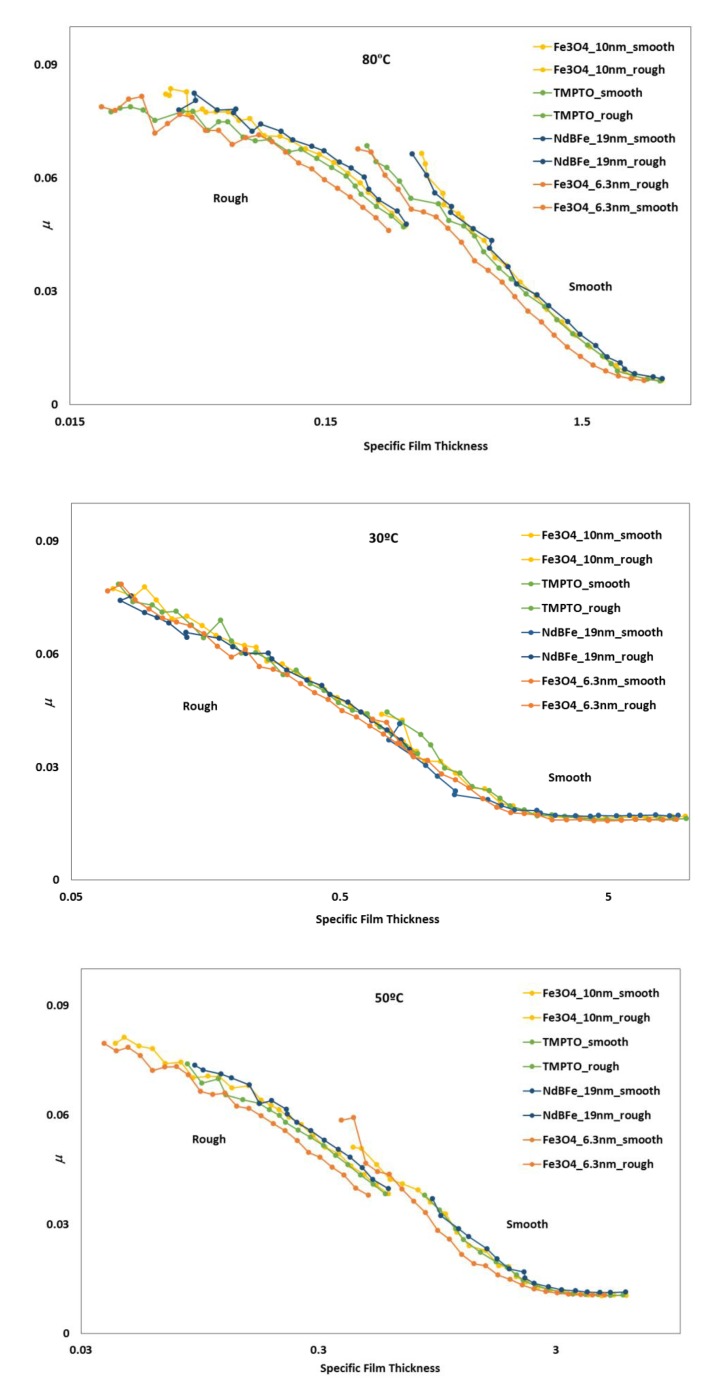
Stribeck curves of the base oil and dispersions with magnetic nanoparticles tested for rough and polished discs at 5% SRR.

Full Stribeck curves (Figure 16) were obtained for nanolubricants and base oil: boundary to mixed (for rough disc) and full film lubrication (smooth disc). As expected, the friction tests made with rough discs produced higher friction values than those made with smooth discs. At operating temperature of 30 °C it can be observed that the addition of the studied nanoparticle hardly changed the coefficient of friction of the nanolubricants in comparison with the TMPTO base oil, and there is no difference since viscosity of all dispersions is very similar. As temperature increases, at 50 and 80 °C the Fe_3_O_4_ (6.3 nm) nanolubricant shows smaller friction than the base oil for both polished and rough discs, in accordance with the slightly higher film thicknesses (Figure 16). This result is mainly interesting for oil engine applications, habitually operating at high temperatures (around 100 °C) [49]. Considering these facts, the tribological behavior of the Fe_3_O_4_ (6.3 nm) nanolubricant was also analyzed in a real application of rolling bearings (Section 3.6).

### 3.6. Friction Behavior: Rolling Bearing Test Rig

**Figure 17:**
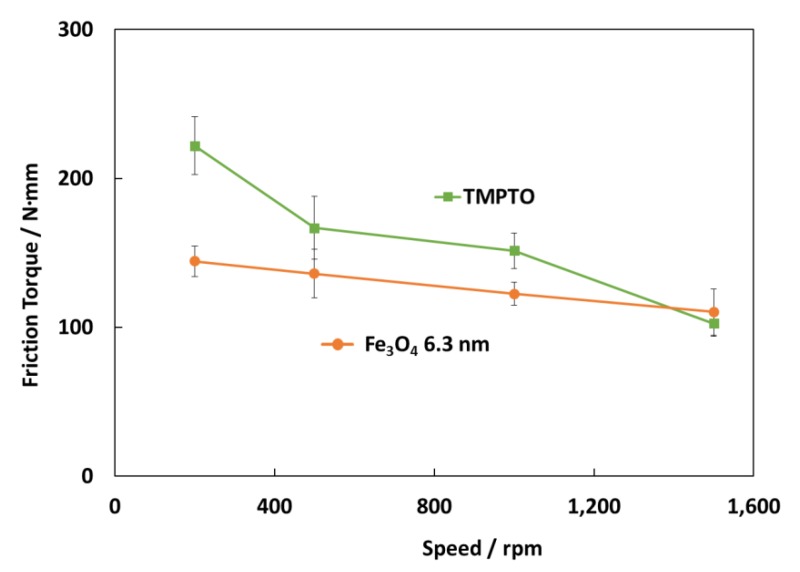
Friction torque results for the rolling bearing lubricated with the TMPTO base oil and TMPTO + 0.015 wt% Fe_3_O_4_ (6.3 nm) nanolubricant.

Figure 17 shows the friction torque results for rolling bearings lubricated with TMPTO base oil and Fe_3_O_4_ (6.3 nm) nanolubricants at 70 °C under 7000 N. The surface roughness of the cylindrical roller thrust bearing is 0.14 μm so that, considering Equation (3), tests were performed under boundary lubrication conditions (*Λ* ≈ 0.06) for both lubricants. The central film thickness (*h*_0_) at this temperature has been predicted through Hamrock and Downson’s equation for elliptical contacts [50]. As Figure 17 shows, friction torque values obtained for the nanolubricant with Fe_3_O_4_ (6.3 nm) at 70 °C are much lower than those for TMPTO base oil, especially at very low speed (200 rpm) likely due to the fact that at boundary conditions the nanoparticles play an anti-friction and anti-wear role. As the speed is increased (500 rpm and 1000 rpm) the behavior is still better than that for the base oil but not as much as for the lowest speed. Finally, at the highest speed (1500 rpm) there is a hardly difference in the friction torque between both lubricants due to the thick lubricant film. The friction torque reduction is about 35% for the first studied speed (200 rpm), 23% for both the speeds of 500 rpm and 1000 rpm.

## 4. Conclusions

In this work the following features were achieved:

- Magnetic nanoparticles: Fe_3_O_4_ (6.3 nm), Fe_3_O_4_ (10 nm) and Nd alloy (19 nm) functionalized with oleic acid have been synthesized.
- Dispersions of TMPTO base oil with 0.015 wt% of Fe_3_O_4_ (6.3 nm), Fe_3_O_4_ (10 nm) and Nd alloy (19 nm) were prepared showing that through chemical modification with oleic acid, a greater stability of the nanodispersions is achieved, which can validate their use for many industrial applications. This is one of the highest time stabilities in the literature for nanolubricants [15].
- For pure sliding/boundary tests at 20 °C, for the three nanolubricants the friction coefficient is lower than that obtained with the base oil, the best friction behavior being obtained with the Nd alloy nanolubricant with a friction reduction of 29% in comparison with the base oil.
- The diameters and depths of the wear scar obtained with the three nanolubricants are lower than those corresponding to the base oil, obtaining maximum wear reductions for the Nd alloy nanolubricant, being 67% and 35% in terms of diameter and depth of the wear scar, respectively
- Protective tribofilm formation was confirmed by confocal Raman microscopy on the worn surfaces.
- Film thickness values for all studied nanolubricants are very similar due to their similar viscosities, so lubrication capacity will be analogous for all lubricants.
- Under rolling conditions of 5% SRR and 30 °C, the full Stribeck curves for all lubricants are similar whereas at the higher temperatures the Fe_3_O_4_ (6.3 nm) nanolubricant shows lower friction coefficient than the base oil and the other nanolubricants.
- Fe_3_O_4_ (6.3 nm) nanolubricant leads to a lower friction torque in comparison with the base oil, especially at low speed when the film is thin and the nanoparticles play an important role in the reduction of friction.

## Figures and Tables

**Table 1:** Oleic acid content in each synthesized nanoparticle.

Sample	Oleic Acid Content (wt%)	Number of Oleic Acid Molecules per NP Surface Area in nm^2^
Fe_3_O_4_@OA (6.3 nm)	29.10	5
Fe_3_O_4_@OA (10 nm)	19.30	6
Nd alloy	14.45	1

**Table 2:** Main physical properties of trimethylolpropane trioleate (TMPTO) base oil and the three nanodispersions of TMPTO with 0.015 wt% of each coated nanoparticle (NP).

Physical Property	TMPTO Base Oil	0.015 wt% (Fe_3_O_4_-6.3 nm)	0.015 wt% (Fe_3_O_4_-10 nm)	0.015 wt% (Nd alloy-19 nm)
Density at 293.15 K/g cm^−3^	0.9161	0.9172	0.9167	0.9177
Viscosity at 313.15 K/mPa s	45.32	46.04	45.81	46.22

**Table 3:** Main characteristics of the ball and discs.

Parameters	Ball	Glass Disc	Steel Disc	
			Polished	Rough
Elastic modulus/GPa	210	75	210	
Poisson ratio	0.29	0.20	0.29	
Radius/mm	19.05	50	50	50
Surface roughness, Ra/nm	20	5	50	500

**Table 4:** Mean values of friction coefficient (*µ*) and of the diameter (wear scar diameter, WSD), depth (wear track depth, WTD) and volume of the wear hole and their respective standard deviations (*σ*) for all nanolubricants for the TMPTO base oil and the three nanolubricants with 0.015 wt% of each NP.

Lubricant	*µ*	*σ*	WSD/μm	σ/μm	WTD/μm	*σ*/μm	Vol/10^3^μm^3^	*σ*/10^3^μm^3^
Neat TMPTO	0.094	0.006	358	15	1.57	0.11	58.1	3.4
Fe_3_O_4_ (6.3 nm)	0.090	0.005	226	17	1.52	0.21	23.7	1.1
Fe_3_O_4_ (10 nm)	0.078	0.005	145	17	1.17	0.13	13.7	0.8
Nd alloy (19 nm)	0.067	0.006	119	13	1.02	0.11	0.56	0.5

**Table 5:** Roughness parameters, *Ra*, and their uncertainties *σ* of worn surfaces lubricated with the three studied nanodispersions and neat TMPTO.

Lubricant	*Ra*/nm	*σ*/nm	Gaussian Filter/mm
TMPTO	60.9	5.2	0.08
TMPTO + 0.015 wt% Fe_3_O_4_ (6.3 nm)	47.9	4.1	0.08
TMPTO + 0.015 wt% Fe_3_O_4_ (10 nm)	41.8	4.2	0.08
TMPTO + 0.015 wt% Nd alloy (19 nm)	37.3	3.8	0.08

## Supplementary Materials

The following are available online at https://www.mdpi.com/2079-4991/10/4/683/s1: Figure S1: Chemical structure of trimethylolpropane trioleate, Figure S2: Raman spectrum of trimethylolpropane trioleate (TMPTO), Figure S3: Raman spectrum of both Fe_3_O_4_ (6.3 nm) and Fe_3_O_4_ (10 nm) nanoparticles coated with oleic acid, Figure S4. Raman spectrum of Nd alloy (19 nm) nanoparticles coated with oleic acid. Table S1: Experimental density, *ρ*/g⋅cm^−3^ of the nanodispersions and base oil as a function of temperature, Table S2: Experimental dynamic viscosity, *η*/mPa⋅s of the nanodispersions and base oil as a function of temperature.
