# Supplementary material for: Tribological Behavior of Nanolubricants Based on Coated Magnetic Nanoparticles and Trimethylolpropane Trioleate Base Oil
Source: Nanomaterials (Basel). 2020 Apr 5;10(4):683. doi: 10.3390/nano10040683 (PMC7221784; doi:10.3390/nano10040683)
Supplement: Supplementary file 1 [file nanomaterials-10-00683-s001.pdf]

Supplementary Material

Tribological Behavior of Nanolubricants Based on Coated Magnetic Nanoparticles and Trimethylolpropane Trioleate Base Oil

José M. Liñeira del Río [1], Enriqueta R. López [1], Manuel González Gómez [2], Susana Yáñez Vilar [2], Yolanda Piñeiro [2], José Rivas [2], David E. P. Gonçalves [3], Jorge H. O. Seabra [4] and Josefa Fernández [1,*]

1. Laboratory of Thermophysical Properties, Nafomat Group, Department of Applied Physics, Faculty of Physics, Universidade of Santiago de Compostela, 15782, Santiago de Compostela, Spain; josemanuel.lineira@usc.es (J.M.L.); enriqueta.lopez@usc.es (E.R.L.)
2. Applied Physics Department, NANOMAG Laboratory, Faculty of Physics, Universidade de Santiago de Compostela (USC), 15782 Santiago de Compostela, Spain; manuelantonio.gonzalez@usc.es (M.G.G.); susana.yanez@usc.es (S.Y.V.); y.pineiro.redondo@usc.es (Y.P.); jose.rivas@usc.es (J.R.)
3. Institute for Science and Innovation in Mechanical Engineering and Industrial Engineering (INEGI), Universidade do Porto, Dr. Roberto Frias St., 4200-465 Porto, Portugal; degoncalves@inegi.up.pt
4. Faculty of Engineering of the University of Porto (FEUP), Dr. Roberto Frias St., 4200-465 Porto, Portugal; jseabra@inegi.up.pt

* Correspondence: josefa.fernandez@usc.es (J.F.)

Received: 27 February 2020; Accepted: 27 March 2020; Published: date

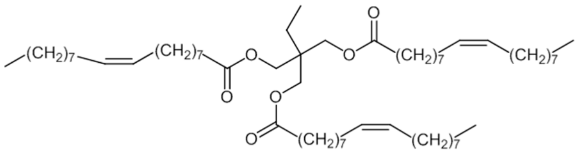

Figure S1. Chemical structure of trimethylolpropane trioleate.

Table S1. Experimental density, *ρ* / g·cm^{-3}of the nanodispersions and base oil as a function of temperature.

T/K	*ρ* / g cm^{-3}			
	TMPTO	*0.015 wt% (Fe_3O_4-6.3nm)*	*0.015 wt% (Fe_3O_4-10 nm)*	*0.015 wt% (Nd alloy -19 nm)*
278.15	0.9258	0.9270	0.9264	0.9274
283.15	0.9226	0.9238	0.9234	0.9242
288.15	0.9193	0.9204	0.9197	0.9209
293.15	0.9161	0.9172	0.9167	0.9177
298.15	0.9129	0.9137	0.9132	0.9144
303.15	0.9096	0.9106	0.9101	0.9112
308.15	0.9064	0.9071	0.9068	0.9079
313.15	0.9031	0.9037	0.9034	0.9047
318.15	0.8998	0.9007	0.9001	0.9014
323.15	0.8966	0.8974	0.8970	0.8981
328.15	0.8933	0.8940	0.8937	0.8949
333.15	0.8900	0.8907	0.8903	0.8915
338.15	0.8867	0.8875	0.8871	0.8883
343.15	0.8835	0.8842	0.8840	0.885
348.15	0.8802	0.8810	0.8807	0.8817
353.15	0.8769	0.8775	0.8772	0.8784
358.15	0.8736	0.8743	0.8740	0.8751
363.15	0.8704	0.8712	0.8707	0.8719
368.15	0.8671	0.8678	0.8674	0.8686
373.15	0.8639	0.8644	0.8642	0.8653

Table S2. Experimental dynamic viscosity, η / mPa·s, of the nanodispersions and base oil as a function of temperature.

T/K	η / mPa·s			
	TMPTO	*0.015 wt% (Fe_3O_4-6.3nm)*	*0.015 wt% (Fe_3O_4-10 nm)*	*0.015 wt% (Nd alloy -19 nm)*
278.15	247.3	252.4	251.0	254.3
283.15	184.2	187.9	186.7	189.1
288.15	140.1	142.8	141.9	143.6
293.15	108.4	110.4	109.8	111.1
298.15	85.29	86.83	86.34	87.25
303.15	68.14	69.32	68.95	69.63
308.15	55.21	56.14	55.84	56.36
313.15	45.32	46.04	45.81	46.22
318.15	37.64	38.23	38.05	38.36
323.15	31.61	32.11	31.96	32.21
328.15	26.82	27.15	27.02	27.30
333.15	22.98	23.24	23.14	23.27
338.15	19.84	20.10	20.02	20.13
343.15	17.28	17.53	17.44	17.54
348.15	15.15	15.36	15.29	15.37
353.15	13.38	13.55	13.49	13.56
358.15	11.88	12.04	11.98	12.04
363.15	10.62	10.75	10.70	10.75
368.15	9.536	9.646	9.607	9.650
373.15	8.611	8.714	8.675	8.714

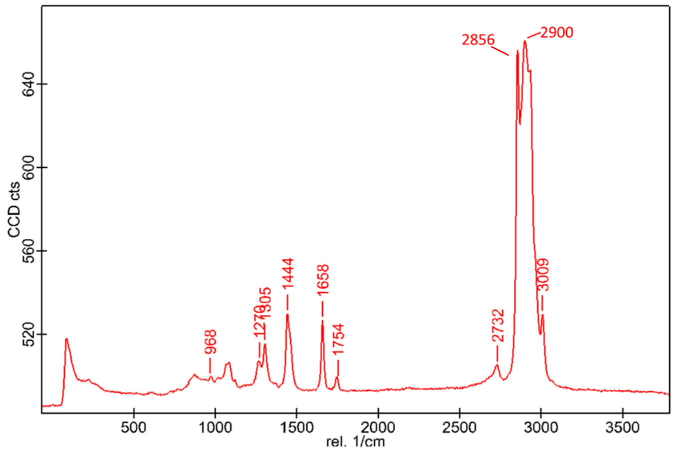

Figure S2. Raman spectrum of trimethylolpropane trioleate (TMPTO).

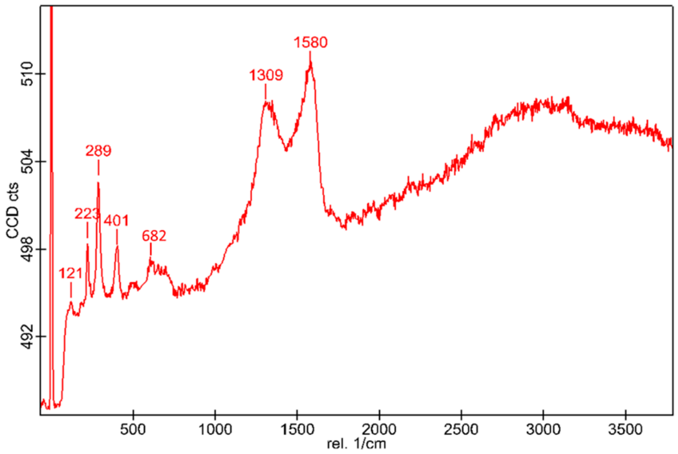

Figure S3. Raman spectrum of both Fe_3O_4 (6.3nm) and Fe_3O_4 (10 nm) nanoparticles coated with oleic acid.

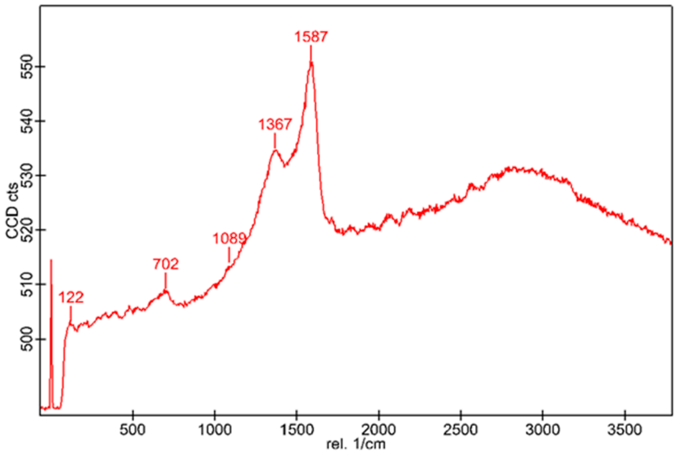

Figure S4. Raman spectrum of Nd alloy (19 nm) nanoparticles coated with oleic acid.
